# Effects of silicon doping on low-friction and high-hardness diamond-like carbon coating via filtered cathodic vacuum arc deposition

**DOI:** 10.1038/s41598-021-83158-4

**Published:** 2021-02-11

**Authors:** Jae-Il Kim, Young-Jun Jang, Jisoo Kim, Jongkuk Kim

**Affiliations:** 1 Surface Technology Division, Department of Extreme Environmental Coatings, Korea Institute of Materials Science (KIMS), 797 Changwondae-ro, Seongsan-gu, Changwon-si, Gyeongnam-do 51508 Republic of Korea; 2 Department of Precision Mechanical Engineering, Kyungpook National University, 2559 Gyeongsang-daero, Sangju, Gyeongsangbuk-do 37224 Republic of Korea; 3 Department of Advanced Science and Technology Convergence, Kyungpook National University, 2559 Gyeongsang-daero, Sangju, Gyeongsangbuk-do 37224 Republic of Korea

**Keywords:** Mechanical engineering, Materials science

## Abstract

In this study, silicon (Si) was doped on a tetrahedral amorphous carbon (ta-C) coating and the tribological characteristics of the resulting Si-doped diamond-like carbon (DLC; a-C:Si:H) were investigated against a SUJ2 ball. The Si fraction in the coating was varied from 0 to ~ 20 at.% by increasing the trimethylsilane gas flow rate during filtered cathodic vacuum arc deposition. The coefficient of friction (CoF) showed no obvious change when the Si fraction was less than ~ 7 at.%. However, after Si doping, it significantly decreased when the Si fraction was greater than ~ 8 at.%. The running-in period also decreased to less than 1000 cycles after Si doping. The rapid formation of Si-rich debris and transfer layer led to the fabrication of a low-friction tribofilm, which was induced by the tribochemical reaction with moisture under ambient conditions. When the Si fraction was ~ 17 at.%, the lowest CoF of less than 0.05 was obtained. Further Si doping beyond the critical point led to the destruction of the film because of reduced hardness.

## Introduction

Diamond-like carbon (DLC) is a fascinating material as a surface coating material in various industries, such as automobile and machinery, owing to its excellent mechanical properties and tribological characteristics, particularly as a solid lubricant^1^. Among several types of DLC, tetrahedral amorphous carbon (ta-C), which is also categorized as hydrogen-free DLC, has been a research hotspot because it exhibits superior hardness, thermal stability, and wear resistance compared with other DLC coatings^2^. However, the property of high hardness is not always conducive for enhancing the tribological behaviors of driving parts because it can induce more wear of counter parts, thus generating abrasive debris^3^. Furthermore, delamination, a critical problem of hard coatings, easily occurs when the internal stress in the coating exceeds its deformation limit^4–7^. Thus, achieving appropriate mechanical properties for the adhesion stability of DLC coatings is crucial. In terms of tribological characteristics, the formation of low-friction debris and corresponding tribofilm via tribochemical reactions plays more important role to achieve low friction and wear compared with the hardness of the coating material itself^8–10^.

To satisfy these requirements, the use of various dopants in DLC coatings has been widely studied^11–15^. When dopants are added to DLC, the internal stress can be reduced, which enhances the adhesion of the coating^16–18^. Moreover, some dopants can lead to the formation of low-friction debris and tribofilms via tribochemical reactions, particularly under lubrication^19–22^. Previous studies on doped DLC coatings have utilized appropriate dopants that match a specific requirement. Fluorine (F) doped on DLC (F-DLC) effectively controls surface energy and provides hydrophobic characteristics to the surfaces^23^. To modify the electrical properties of DLC, nitrogen is doped on DLC by changing the charge transfer mechanism^24–26^. Among metallic materials, tungsten (W) and chromium (Cr) have been used to produce a wear-resistant DLC coating with enhanced mechanical properties^9,21,22^. For biomedical applications, silver-doped DLC (Ag-DLC) has also been investigated for its biocompatibility^27,28^.

Among several dopants, Si and Molybdenum (Mo) are fascinating dopants for tribological applications^29–32^. DLCs coated with these dopants are effective when used under lubricating conditions^33,34^, and Si and Mo are widely used as lubricating oil additives in driving parts. Si-doped DLC has been investigated under water-lubricating conditions^34^, and it can act as a solid lubricant under high humidity^35^. Furthermore, surrounding gas and temperature are important factors affecting the low-friction condition of Si-doped DLC owing to the tribochemical reaction^36^. Si-rich debris worn from the coating can form a silica sol [SiO_x_(OH)_y_] and absorb moisture under ambient conditions. Appropriate temperature regimes can promote the formation of low-friction compounds on the surface^36^. The corresponding Si-rich transfer layers and tribofilms are attributed to low friction and wear. However, the type of transfer layer and tribofilm can vary depending on the counter material. For instance, iron oxide can be easily formed when the DLC coating generates friction and wear with steels because the majority of debris, which can produce a transfer layer and tribofilm via the tribochemical reaction, is composed of steel owing to the considerable difference in hardness^37^. Therefore, to date, ceramic and/or Si-containing counter parts have been used for low-friction Si-doped DLC to suppress the undesired formation of compounds such as iron oxides. Moreover, most studies have used Si dopants on hydrogenated amorphous carbon with hydrogen (a-C:H). The effects of Si doping on ta-C against steels have not yet been explored.

In this study, the effects of Si doping on ta-C coating via filtered cathodic vacuum arc (FCVA) deposition using trimethylsilane (TMS) gas were investigated against the SUJ2 ball. It is important to understand the friction mechanisms against steel because most driving parts in automobiles and machinery are composed of steel. SUJ2 is a widely used material for bearings, which are the target application of this research. The amount of Si dopants was controlled by changing the flow rate of TMS gas, and the optimal Si doping level was obtained based on tribological characteristics. Although the microstructure of the FCVA-deposited film corresponded to that of ta-C, it was transformed into other structures; therefore, the Si-containing DLC films are named Si-doped DLC in this study. The coating was characterized to investigate variations in Si fractions and microstructures. Tribotests were performed using a ball-on-disk experimental setup, and the corresponding friction and mechanism of tribochemical reactions were investigated based on the surface analysis using optical micrographs and scanning electron micrographs. Finally, the optimal coating conditions were established, and the use of the fabricated coating was presented.

## Experimental

### Coating preparation

Tungsten carbide (WC) was used as the substrate material to achieve improved coating adhesion. The surface was ground and polished before coating using SiC papers (180, 320, 600, and 1200 grit) and 1-μm alcohol-based diamond suspension. Then, the substrates were ultrasonically cleaned with deionized water and rinsed with isopropyl alcohol (IPA). To eliminate moisture inside the substrates, they were dried in an oven at 100 °C.

A hybrid coating system comprising a linear ion source (LIS), unbalanced magnetron (UBM) sputter, and FCVA was used for ta-C coating and Si doping. The coating process is typically divided into three steps: (1) surface etching using LIS, (2) interlayer deposition using UBM, and (3) ta-C coating using FCVA. To remove the naturally formed oxide layer and the remaining impurities on the substrates, Ar plasma etching was performed at 2.7 kV and 700 mA using customized LIS. The Ar flow rate was fixed at 40–50 sccm. During the etching process, a substrate bias (*V*_b_) of − 75 V was used. The etching rate was ~ 1 nm/min, and the total etching thickness was ~ 50 nm. The etching and deposition rates were determined by measuring the difference in the thickness after etching and/or coating for an hour by covering only part of the surface. In this study, an interlayer was not used because WC exhibits sufficient adhesion with the coating at the interface. Finally, ta-C was coated via FCVA using a solid graphite target (ex-70, Ibiden co. ltd., *Japan*). Both the diameter and height of the chamber were 800 mm. The diameter of the graphite target was 50 mm. The arc source was constructed using a 90°-bent type duct, and two arc sources were used to achieve a higher deposition rate. The vacuum was exhausted to 0.01 Pa, and TMS gas was injected into the chamber during the FCVA process by varying its flow rate from 0 to 12 sccm. The working pressure was varied from 0.01 to 0.2 Pa during the deposition. The gas was injected into the chamber through the nozzle connected to LIS (Supplementary Figure 1). The turbomolecular pump was placed on top of the chamber to prevent the TMS gas from directly flowing to the graphite target. Therefore, the arc spot motion was not critically affected by the injection of the TMS gas. The cathode voltage was varied between 22 and 24 V to maintain the arc current at 100 A without TMS gas injection. After the TMS gas injection, the cathode voltage was slightly decreased to 21–22 V because a small amount of TMS gas can flow into the duct around the graphite target. The arc and carbon plasma were controlled using a source magnet, extraction magnet, and outlet magnet, as shown in Supplementary Figure 1. Then, the arc was controlled according to the general experimental setup of FCVA methods. During deposition, the temperature inside the chamber was maintained at 120 °C. After the TMS gas injection, the deposition rate was increased compared with that of pure ta-C owing to the additional deposition of carbon and silicon from gas decomposition. Thus, the processing time was controlled to fix the thickness of ta-C and/or Si-doped DLC coating at ~ 1 μm. The schematic and detailed experimental conditions are also summarized in Supplementary Figure 1 and Supplementary Table 1.

**Figure 1:**
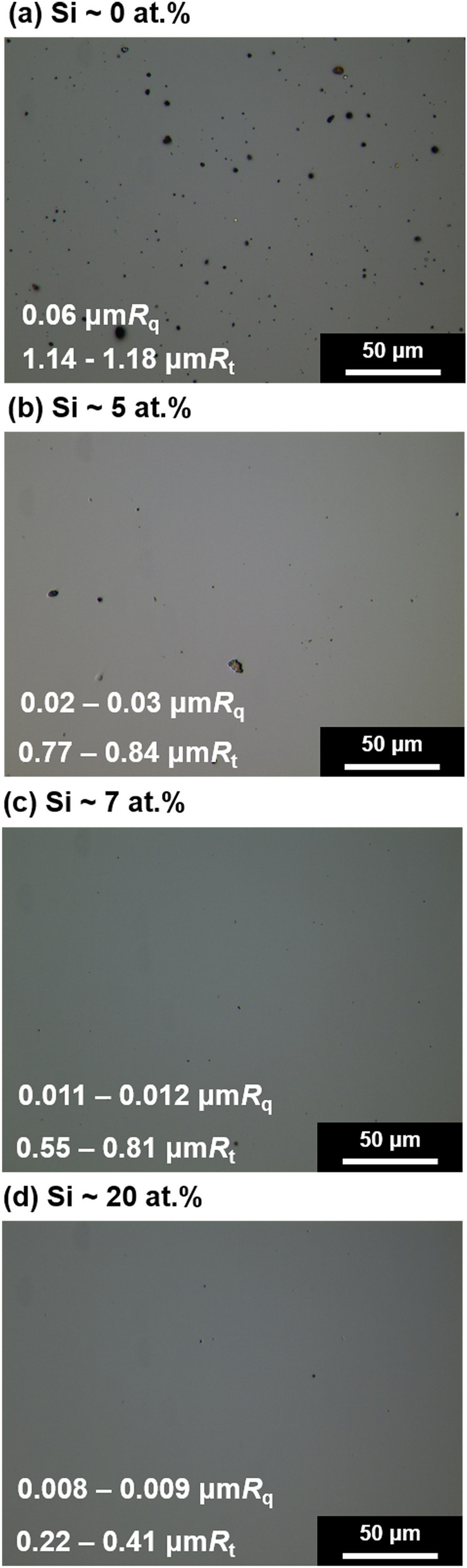
Representative optical micrographs of the surfaces and corresponding roughness values (*R*_q_ and *R*_t_) in terms of Si fractions doped on DLC coatings.

### Coating characterizations

The chemical composition of the coating before and after Si doping was analyzed using two different methods: one was X-ray photoelectron spectroscopy (XPS; K-alpha, ThermoFisher, *USA*) and the other was energy-dispersive X-ray spectroscopy (EDS). XPS analysis was performed using an energy step size and take angle of 0.45 eV and 90°, respectively, on the as-received surface of coated samples to investigate the outermost surface bonds without any pretreatment such as etching. EDS analysis was performed using a 5-keV acceleration voltage to determine the chemical composition of the bulk film. The elemental composition was recorded and analyzed on ESCALAB 250 XI (Thermo Fisher Scientific, *United States*) using Al-Kα radiation. The C 1 s spectra were fitted using the Gaussian–Lorentzian convolution (Voigt) function to specify the sp^2^/sp^3^ ratio of the coated films^38,39^. For the pure ta-C coating, the C 1 s spectra were fitted with the centers of four peaks at 284.4 eV (C–C/C–H; Csp^2^), 285.1 eV (C–C; Csp^3^), 286.6 eV (C–O), and 288.5 eV (C = O). A peak center at 283.3 eV (Si–C) was additionally involved in Si-doped DLC coatings. Aerial fractions under the fitted curves were used to determine the sp^2^ and sp^3^ fraction of each coating. The surface nanohardness was measured using a nanoindenter (NHT3, Anton Paar, *Austria*) with a maximum indentation depth of ~ 100 nm. To minimize the effects of surface roughness and macroparticles on nanohardness, the measurement was performed at least five times in different regions by avoiding macroparticles. Surface roughness of the coated substrates was evaluated using a contact-type surface profilometer (Mitutoyo SJ-410, *Japan*). The contact angles on the coatings were measured by the Sessile-drop method using deionized water with a 5-μL volume.

### Tribological characterization

Ball-on-disk tests were performed to investigate the tribological characteristics of the coated films with and without Si dopant. For the reliability of low-friction coating against bearing steels, a SUJ2 ball (diameter = 8 mm, G20, 62–67 HRC) was used for the tests. Supplementary Table 2 summarizes the chemical composition of the SUJ2 ball. The normal load was 5 N, and the rotating speed was 200 rpm. The tests were performed under ambient conditions (55–60% humidity and 24–26℃ temperature). The radius of the rotating track was 4.5 mm, and the tests were performed for 2 h. The corresponding total rotation and sliding distance were 24,000 cycles and 678.6 m, respectively. During rotation, the coefficient of friction (CoF) was recorded using a strain gauge placed parallel to the substrate surface. Supplementary Figure 2 shows the experimental setup of the ball-on-disk tests. Worn areas on the ball, wear debris, and tribofilms were analyzed using an optical microscope (Leica, *Germany*) and field-emission scanning electron microscopy (NovaNano-230, FEI, *United States*) equipped with EDS. The chemical composition of the wear debris and thin tribofilms was evaluated using EDS analysis. The wear rates were calculated according to the measured wear volumes using a confocal microscope (OLS500, Olympus, *Japan*). To achieve better reliability of measurements, the wear volume was measured at four different areas on the wear track and averaged.

## Results and discussion

### Characterization of Si-doped DLC films

Tribological characteristics such as friction and wear can be affected by abrasion, adhesion, and mechanical interlocking. Among them, mechanical interlocking most intuitively affects friction by inducing physical obstruction. This can be significantly affected by the surface roughness of the coating. Thus, it is important to reduce the surface roughness. Therefore, filtering large particles generated during the carbon deposition process using a magnetic field is an effective approach for reducing the surface roughness. However, the secondary deposition of macroparticles projected from the duct, filters, and side walls of the vacuum chamber cannot be entirely prevented using the FCVA method. Thus, several macroparticles were observed on the surface (Fig. 1a). However, after adding the TMS gas, the number of particles on the film surfaces decreased. The number of particles reduced with an increase in the gas flow rate. When the TMS gas flow rate was increased from 0 to 12 sccm, the roughness decreased from 0.06 μm*R*_q_ to 0.008 μm*R*_q_. Moreover, the peak-to-valley distance (*R*_t_) clearly decreased from 1.14 μm*R*_t_ to 0.22 μm*R*_t_. This reduction in the surface roughness could effectively decrease the abrasive friction and wear, thereby eliminating mechanical interlocking. A decrease in the surface roughness could be attributed to the change in the deposition mechanism and a shorter processing time, which resulted in a higher deposition rate when the TMS gas was used for doping Si on the DLC coating. When FCVA was used without the TMS gas for pure ta-C deposition, ionized carbon plasma was directly deposited on the surface of WC samples. With an increase in the processing time, macroparticles can be generated from the contaminated chamber and ducts, degrading the surface roughness of the coating. However, when coating was performed using the TMS gas, an increased working pressure (Supplementary Table 1) could form a relatively stable plasma of carbon with the simultaneous deposition of fully and/or partially broken Si–CH_3_, –CH_3_, and Si–C components from the TMS molecules. This could suppress the formation of macroparticles during the FCVA deposition. Furthermore, an increased deposition rate enhanced the surface roughness of coatings. The formation of macroparticles increases with the deposition time when the number of plasma ignition processes increases^40^. Moreover, contamination (i.e., carbon coated on the baffles, ducts, and walls of the chamber) can be delaminated to form macroparticles when its thickness exceeds a certain level. Thus, the shortened processing time with a higher deposition rate using the TMS gas could suppress the emission of macroparticles during the deposition process. The crucially reduced *R*_t_ (approximately one fifth level), confirmed the decrease in the formation of macroparticles on the surface after the TMS gas injection.

**Figure 2:**
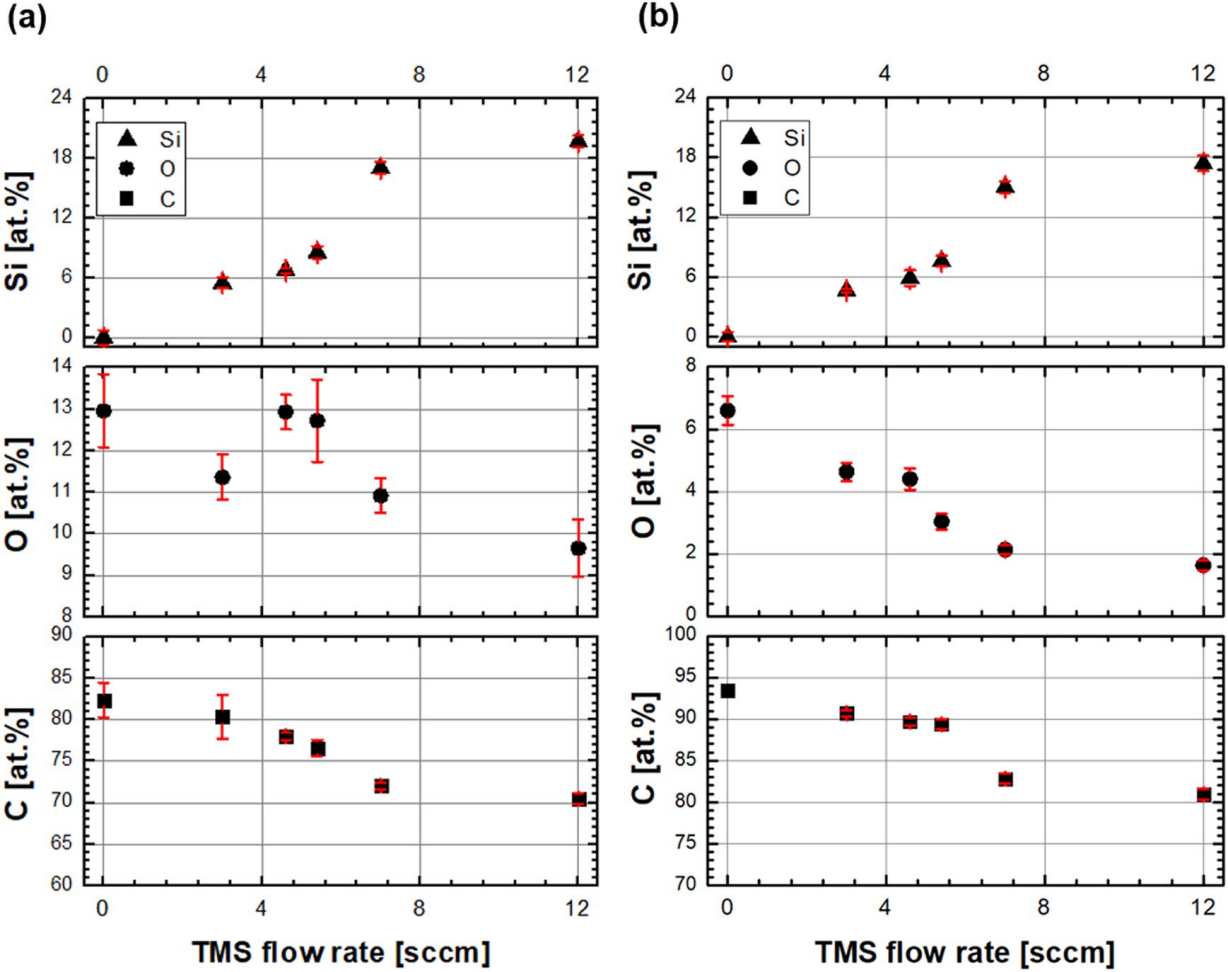
Chemical compositions of Si-doped DLC coatings in terms of TMS gas flow rates analyzed using (**a**) X-ray photoelectron spectroscopy (XPS) and (**b**) energy-dispersive X-ray spectroscopy (EDS).

**Figure 3:**
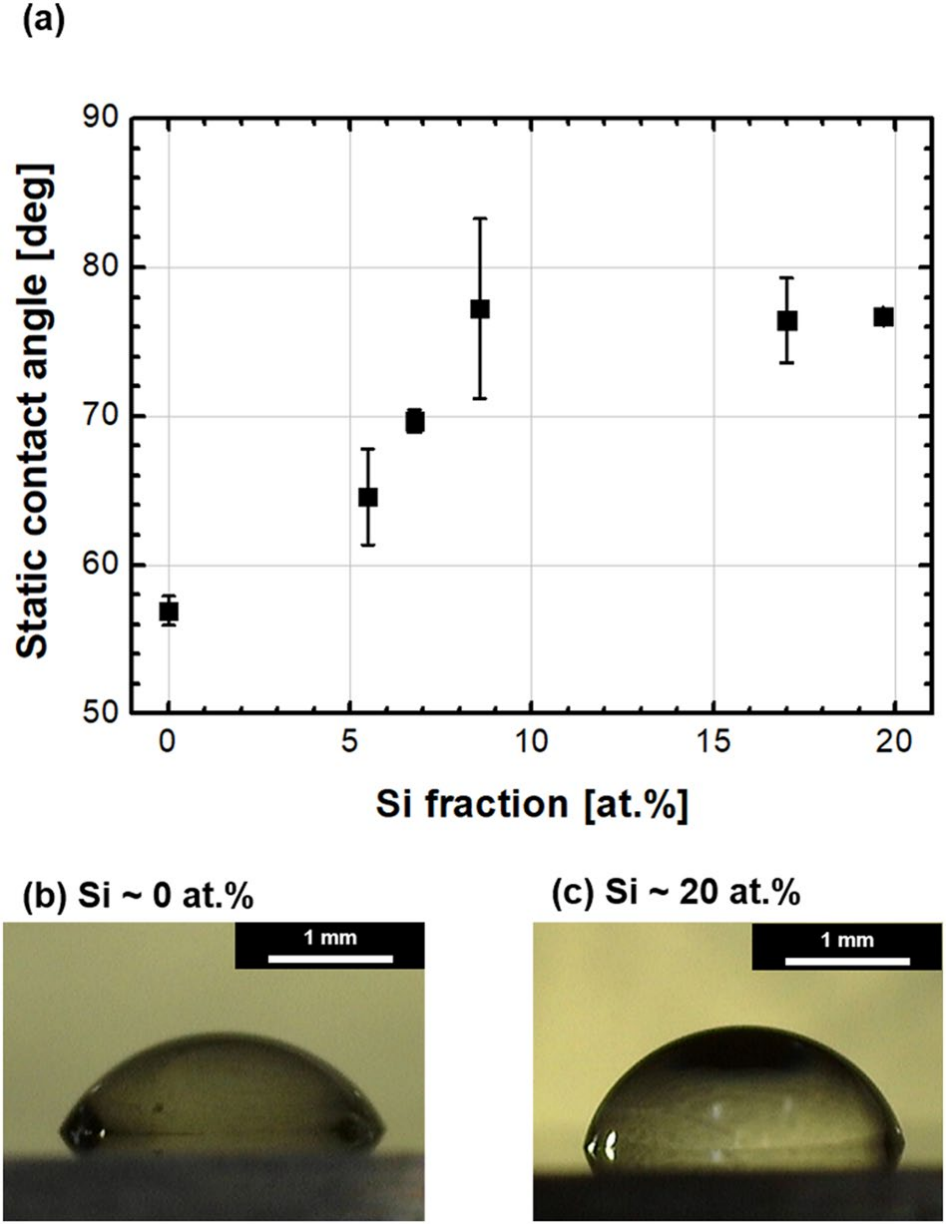
(**a**) Variations in static contact angles and (**b**) and (**c**) representative optical micrographs of droplets in terms of Si fractions.

Figure 2 shows the variation in the chemical compositions of silicon, oxygen, and carbon on the coatings as a function of TMS flow rates. The chemical composition obtained from the XPS spectra represented the composition of the outermost surface owing to a relatively thin penetrating thickness (Fig. 2a). Conversely, the chemical composition obtained from the EDS spectra was related to the chemical composition of the bulk film (Fig. 2b). Although the Si fraction was slightly higher on the outermost surface than that at the bulk, the variation tendency after increasing the TMS gas flow rate well matched between the outermost surface and the bulk films. Because most tribological properties are affected by the characteristics of the outermost surface, the effects of Si doping on the DLC coating were investigated in terms of Si fractions on the outermost surface. The Si fraction on the outermost surface was increased from 0 to ~ 20 at.% by increasing the TMS gas flow rate from 0 to 12 sccm. The increasing Si fraction indicated the successful doping of Si using the TMS gas during FCVA coating. The high energy of carbon plasma was sufficient to decompose Si–C bonding in Si(CH_3_)_4_ molecules, and the decomposed Si and CH_3_ could be simultaneously deposited on the substrates with carbons. Moreover, a decrease in the oxygen fraction with an increase in the TMS flow rate could indicate reduced free-standing dangling bonds on the outer carbon. The ta-C coating deposited using the FCVA method could contain many free-standing dangling bonds after deposition because only ionized carbons were coated on the substrates. Next, these bonds could react with oxygen under ambient conditions when the substrates were extracted from the vacuum chamber. The lower oxygen fraction in the bulk films (Fig. 2b) than in the outermost surface (Fig. 2a) clearly supported this phenomenon. However, the free-standing dangling bonds could be terminated by the decomposed TMS gas, such as Si–, Si–CH_3_, and CH_3_, as indicated by the higher fraction of Si and C on the outermost surface than that in the bulk films. Finally, the surface energy could be lowered by the formation of hydrophobic surface functional groups instead of remaining free-standing dangling bonds, which exhibit relatively high surface energy. Figure 3 shows the variations in the static contact angles measured on the coated surfaces in terms of Si fraction. The result verified the decrease in the surface energy after increasing the Si fraction. Thus, reduced surface energy can also affect the friction characteristics of coatings with a decrease in the adhesive friction behaviors.

**Figure 4:**
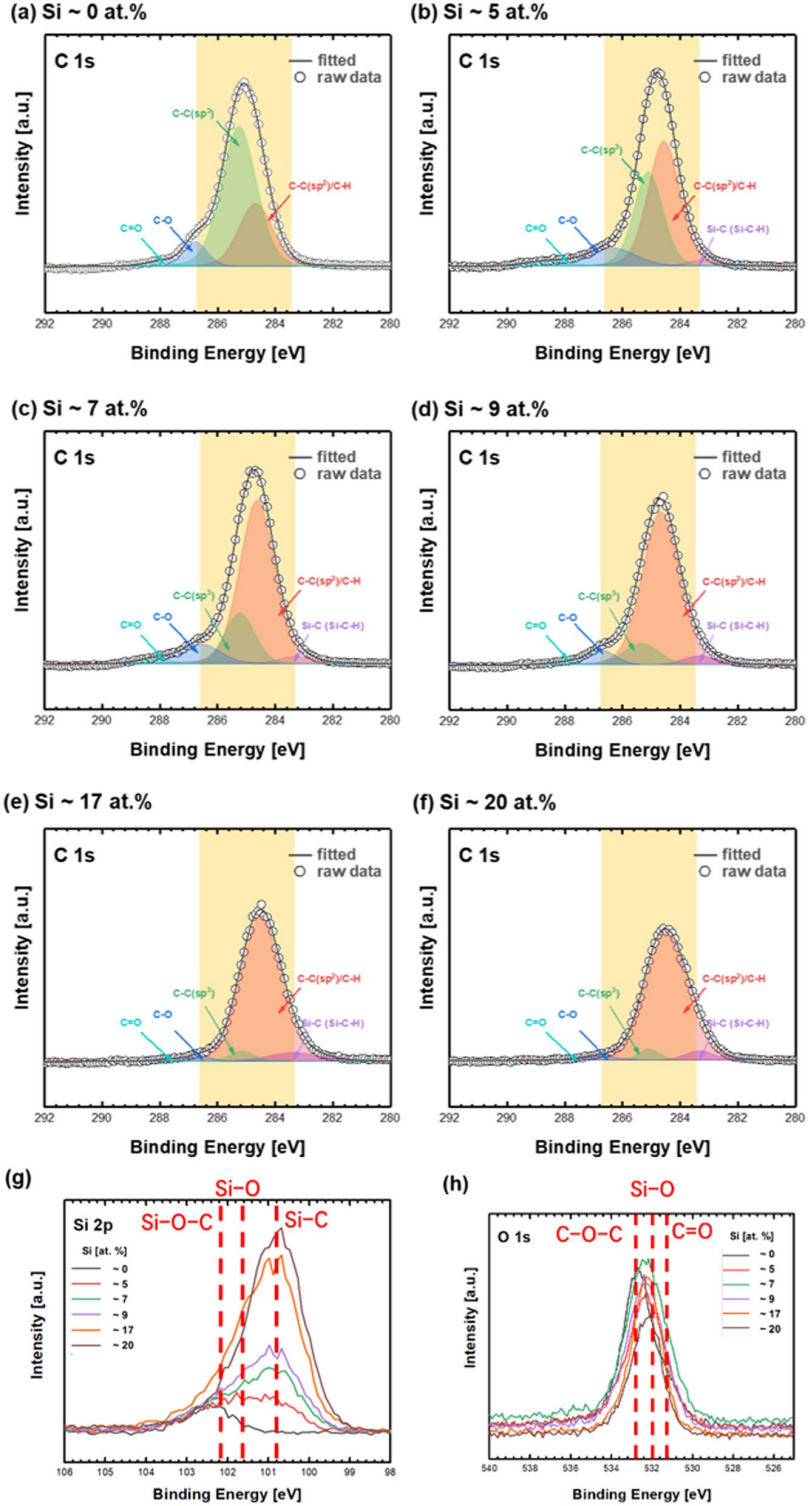
(**a**–**f**) C 1 s, (g) Si 2p, and (**h**) O 1 s XPS spectra in terms of Si fractions doped on DLC coatings with fitted lines (indicated by the solid line).

Both the surface topology and microstructure of the coatings changed after Si doping. Figure 4a–f show the C 1 s XPS spectra of pure ta-C and Si-doped DLC using the TMS gas during the deposition process. As shown in Fig. 4a, the original microstructure of ta-C mainly comprised sp^3^, indicating the tetrahedral structure of the amorphous carbon film. The mixed Gaussian–Lorentzian fitting of the spectra well matched the original line using the four centers of each peak. When a very small amount of Si doping was used (~ 5 at.%), the XPS spectra revealed a stretched shoulder near 283.3 eV, suggesting the emergence of Si–C/Si–C–H bonds. This observation was likely attributable to the reaction between carbon atoms and decomposed TMS gas. The aerial fraction of Si–C/Si–C–H bonds increased when the Si composition in the films increased. Furthermore, the fraction of sp^3^ continuously decreased with an increase in the Si dopants. When the Si fraction was increased, the peak shift of Si 2p spectra also matched the tendency of C 1 s spectra. A decrease in the Si–O–C bonds and an increase in the Si–C bonds were observed in the spectra (Fig. 4g). Furthermore, the decreasing tendency of Si–O–C could confirm the decreased free-standing dangling bonds on Si-doped DLC, which could react with oxygen after the deposition process. The TMS gas flow rate effectively terminated the free-standing dangling bonds by the reaction of these bonds with Si to form Si–C, as indicated by the sharp peak at 100.9 eV in Fig. 4g. The XPS spectra of O 1 s also indicated a peak shift after a decrease in the C–O–C bonds. However, the most important observation in O 1 s spectra was the decrease in intensity with an increase in Si fraction. This finding well supports the claim that free-standing dangling bonds could be terminated using the decomposed TMS gas and prevent the formation of oxides on the surface.

**Figure 5:**
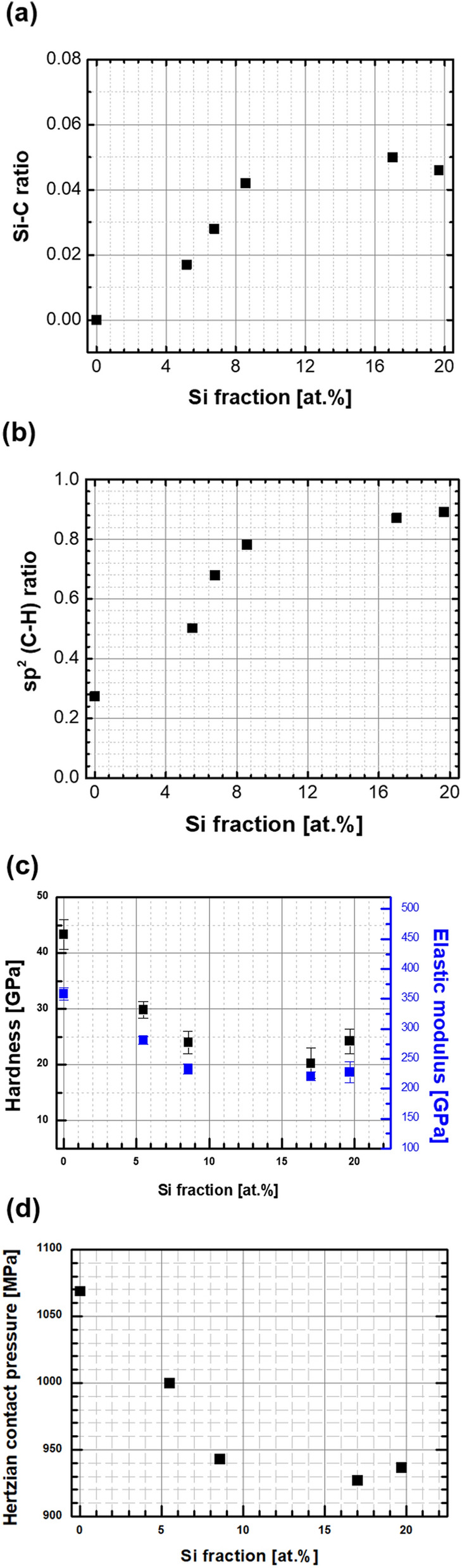
(**a**) Si–C and (**b**) sp^2^ (C–H) ratio of the coatings in terms of Si fractions analyzed using XPS, and variations in **(c**) nanohardness, elastic modulus, and (**d**) corresponding Hertzian contact pressure against the SUJ2 ball in terms of Si fractions.

Figure 5a, b show the Si–C and sp^2^ ratio of the coatings with and without Si doping. The representative ratios were calculated using the aerial fraction of fitted curves from Fig. 4. The sp^2^ ratio of the coating with Si doping significantly increased compared with that of the ta-C coating, i.e., the ratio increased from 0.27 to 0.87 when the Si fraction was increased to ~ 17 at.%. The sp^2^ ratio saturated near 0.9 when the Si fraction was greater than ~ 17 at.%. Thus, Si-doped films cannot be categorized as ta-C:Si. The increasing sp^2^ ratio could project a decrease in the mechanical properties, particularly hardness. Figure 5c, d show the variation in the hardness and Hertzian contact pressure on the coated surface after the Si fraction was increased. Both nanohardness and elastic modulus decreased to ~ 20 and ~ 230 GPa, respectively. Although the Hertzian contact pressure decreased with an increase in the Si fraction owing to the decreased elastic modulus, most values at 1 GPa were sufficient to estimate the tribological application of the coating for bearing applications not exceeding fatigue limits^41^. In many previous studies on Si-doped DLC coatings, the addition of dopants could decrease the hardness and modulus because interstitial atoms or molecules destroyed the substantial and stable microstructures of coated layers^42^. Conversely, the hardness increased after doping when the original hardness of the coated layer was very low^43^. However, in most cases, hardness could not exceed ~ 20 GPa because doping was performed on hydrogenated amorphous carbon (a-C:H) and/or hydrogen-free amorphous carbon with a relatively high sp^2^ ratio (a-C)^11,42–45^. In case of Si doping on DLC coating using TMS gas, the hardness remained above 20 GPa, although the fraction of sp^2^ critically increased. A previous study revealed that the hardness of Si-doped DLC could be maintained or slightly increased with an increase in the Si fraction^46^ if the fraction of hydrogen was lowered with increasing Si fraction. Moreover, the fraction of hydrogen could be lowered when Si deposition was performed using magnetron sputtering with a solid silicon carbide target^46^. However, Si doping in this study was performed using the TMS gas, implying that Si doping could be achieved in the form of Si– and Si–CH_3_. The fraction of hydrogen in the coating could be increased by increasing the TMS gas flow rate during the FCVA process; therefore, increasing the Si fraction clearly decreased the hardness of the coating. Thus, in this study, the structure of Si-doped DLC was transformed from ta-C into a-C:Si:H rather than ta-C:Si. The wear rate increased with a decrease in the hardness of the coated surface.

### Tribological characteristics

**Figure 6:**
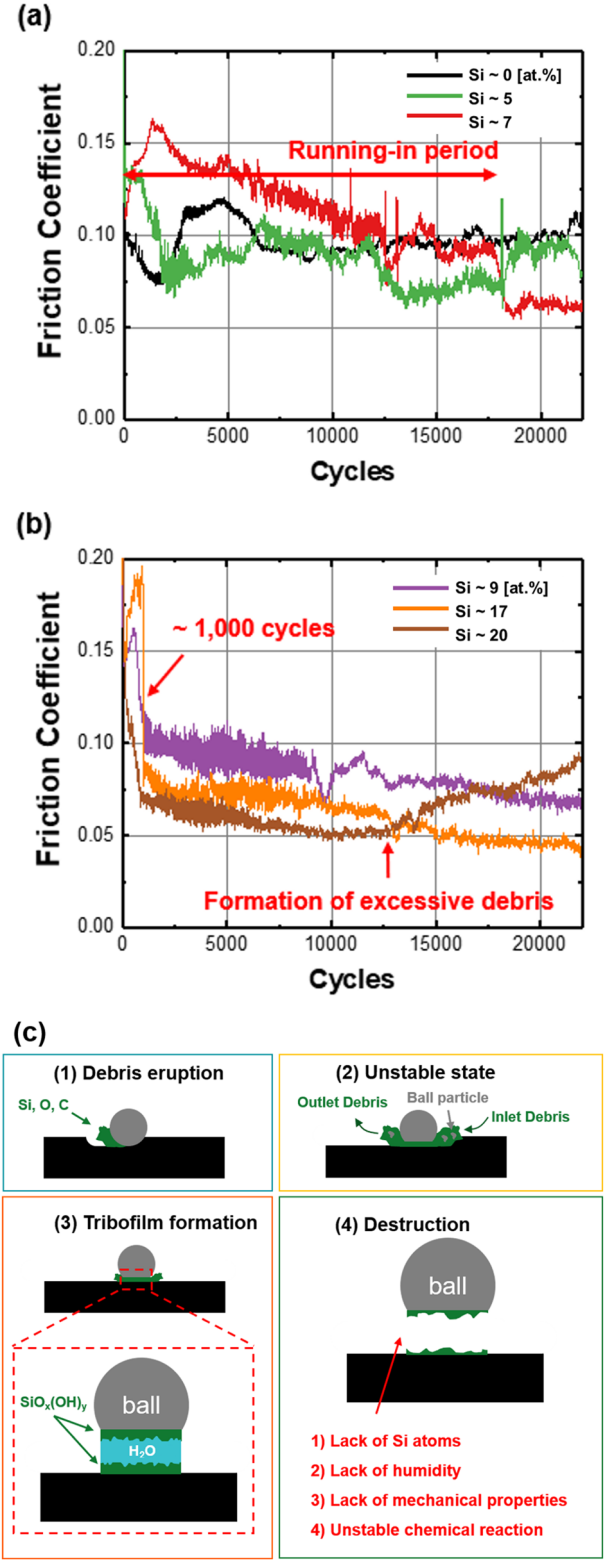
Variations in the coefficient of friction (CoF) during ball-on-disk tests for the Si fraction of (**a**) less than ~ 7 at.% and (**b**) greater than ~ 8 at.%, and schematic diagrams describing the friction mechanisms on Si-doped DLC coatings under ambient conditions with humidity.

The tribological characteristics of coated films are affected by the material characteristics of coatings and counterparts, types of wear debris, environment of wear, and corresponding tribofilms formed during friction motions. Figure 6 shows the variation in the CoF of the films with and without Si doping during ball-on-disk tribotests using a SUJ2 counter ball. The CoF of pure ta-C film was maintained at approximately 0.10, which fluctuated considerably during the testing periods. For some time, the value remained stable; however, it again fluctuated continuously regardless of the rotating cycle. Thus, in the graph, it was difficult to identify the running-in period, which corresponds to the transient range of CoF values. The running-in period must be short because most wear can occur in this period. Additionally, after the running-in period, the CoF value generally becomes low and stable because the formation and elimination rate of tribofilm is in the steady state. Figure 6a shows that a small amount of Si doping did not effectively yield low CoF and short running-in period during approximately 24,000 cycles. This figure indicates that when the Si fraction was lower than ~ 7 at.%, the CoF value continued to fluctuate considerably. Although the CoF of the coating was 0.09 when the Si fraction was ~ 5 at.% after the running-in period, more than 17,000 cycles were required to continuously decrease CoF from ~ 0.15. The unstable variation in the CoF of the coating was attributed to several macroparticles, high surface energy, and the formation of abrasive and/or adhesive wear debris such as FeO, Fe_2_O_3_, and Fe_3_O_4_. When DLC coatings were rubbed using a SUJ2 ball, the ball mostly underwent wear owing to a considerable difference in hardness. Iron atoms in worn debris could easily form iron oxides, which are abrasive and adhesive compounds. When the Si fraction was insufficient, an unstable 3-body abrasion resulted in CoF fluctuation with repeated formation and decomposition of debris. Therefore, a stable and low-friction tribofilm was challenging to achieve. For the pure ta-C coating, a rough surface (Fig. 1) and relatively high surface energy (Fig. 3) could cause rapid wear of the counter ball and form abrasive/adhesive wear debris. Consequently, the different tribological behaviors shown in Fig. 6a were mainly attributed to the differences in surface energy and roughness.

Alternatively, during the ball-on-disk tests, the variations in CoF clearly differed for Si-doped DLC coatings when the Si fraction was greater than ~ 8 at.%. As shown in Fig. 6b, CoF initially increased to greater than 0.15 and suddenly decreased after approximately 1000 rotation cycles. Figure 5c shows that the decreased hardness after Si doping could induce a higher amount of worn debris during the initial stage of wear tests. Thus, a large amount of debris generated from coatings can disturb the rotation by acting as an abrasive and/or mechanical interlocking. However, after 1000 cycles, a decrease in CoF was observed due to the formation of stable and low-friction tribofilms. The CoF of less than 0.1 was achieved, which continuously decreased with an increase in the number of rotation cycles. When the Si fraction was higher, CoF decreased rapidly and exhibited a lower level. Furthermore, the fluctuating amplitude in a single cycle, which can be indicated by the width of each graph, decreased when the Si fraction was increased from ~ 9 to ~ 20 at.%. Finally, the CoF of less than 0.05 was achieved, which indicated a considerably enhanced friction behavior at a Si fraction of greater than ~ 17 at.%. A CoF value could be achieved likely because of the formation of Si-rich tribofilms under ambient conditions. Generally, Si atoms form a silica sol that reacts with humid air, as shown in Eq. (1).

1 $$ {\text{ta-C:Si}} + {\text{H}}_{2} {\text{O}} \to {\text{SiO}}_{x} ({\text{OH}})_{y} $$

The formation of silica sol can promote lubricating friction at the interface between the coating and SUJ2 ball because silica sol contains moisture with high surface energy. During the repeated rubbing of SUJ2 ball, the temperature increased and silica sol could be formed when the temperature was sufficiently high to result in a tribochemical reaction. This finding agrees well with the previous investigation on tribological characteristics of Si-doped DLC at high temperatures^36^. For the tribochemical reaction that forms a stable and low-friction tribofilm, a certain level of energy (high temperature in most cases) and a sufficient amount of Si are required. Thus, the temperature that can lead to the tribochemical reaction was reached after approximately 1000 cycles of ball-on-disk rotation. Owing to the formation of silica sol, the Si-doped DLC coating exhibited extremely low CoF in humid air. However, when the Si fraction was excessively high (~ 20 at.%), CoF showed an increasing trend after approximately 12,000 cycles. This finding is attributed to the decreased hardness and the corresponding excessive formation of wear debris. As indicated in the schematic diagram (Fig. 6c), the formation and elimination of wear debris inside the interface should be maintained at a steady state to stabilize the tribofilm on both the ball and wear track. Otherwise, the low-friction tribofilm will be destructed owing to several reasons such as a lack of Si atoms, humidity, mechanical properties, and unstable chemical reaction. Thus, ~ 17 at.% of Si was concluded to be the optimal Si doping level to achieve low friction and long-term durability. Although the coating with ~ 20 at.% Si dopants exhibited the minimum CoF, the average CoF corresponded to the minimum value with ~ 17 at.% Si dopants owing to the continuously increasing CoF after approximately 12,000 cycles with ~ 20 at.% Si dopants.

**Figure 7:**
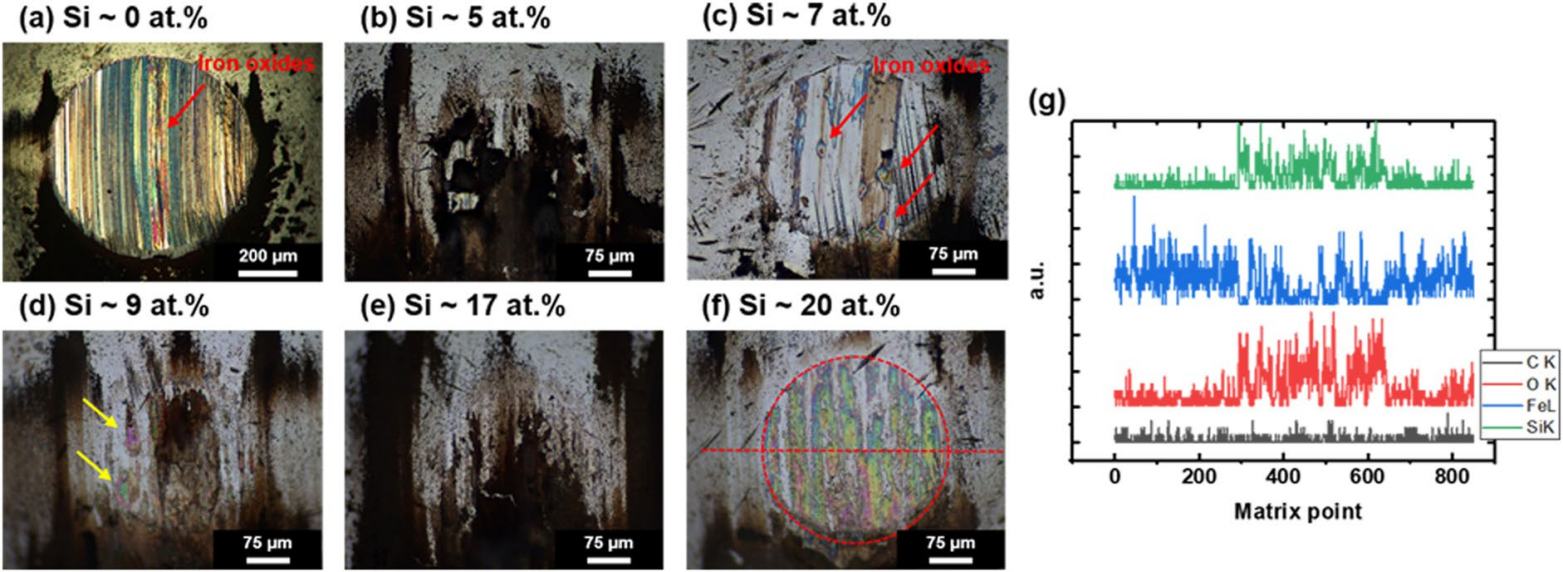
(**a**–**f**) Optical micrographs of worn balls after tribotests in terms of Si fractions and (**g**) line profiling results of energy-dispersive X-ray spectroscopy (EDS) on the wear area along the red-dashed line indicated in (**f**).

Optical micrographs of the ball at worn areas confirmed the formation of stable tribofilms with the optimal Si doping level. As shown in Fig. 7a, the amount of wear generated by the pure ta-C coating considerably higher, as indicated by the longer radius of the wear area. A large amount of worn debris was observed before the formation of tribofilms using a small Si fraction (Fig. 7b). Red-colored iron oxides were clearly observed after the tribotest (Fig. 7a, c), implying that iron oxides were constantly generated and adhered to the surface of the worn ball, which increases CoF inducing 3-body abrasion. In case of pure ta-C, the considerable fluctuation in CoF was attributed to the formation of iron oxide debris, which is verified in Fig. 7a; the figure shows that most of the worn surfaces were covered by the attached iron oxides. In case of Si-doped DLC, the radius of the wear area was considerably shorter than that of the ball worn by pure ta-C, which resulted from the softer counterpart (Si-doped films) compared with the ball. The trace of iron oxide formation was still observed on the ball with a small amount of Si dopants. When the Si fraction was greater than ~ 8 at.%, Si-rich tribofilms (an iridescent area in Fig. 7d) were formed on the surface after the tribotest. However, worn debris composed of Si and O [black- or brown-colored areas in Fig. 7e] did not remain on the wear circle. Low-friction debris was clearly observed on the ball after the tribotest using ~ 17 at.% Si dopants (Fig. 7e). However, only thin Si-rich tribofilms remained on the ball in the case of ~ 20 at.% Si dopants (Fig. 7f). The iridescent film exhibited a very thin thickness of the remaining tribofilm without lubricating debris. EDS analysis of the debris and thin film revealed the formation of Si-rich tribofilms (Fig. 7g). Furthermore, XPS spectra verified the formation of silica sol as shown in Supplementary Figure 3. To confirm the bulk characteristics of Si-doped DLC, XPS analysis was performed after the 200-nm etching on the as-coated surface. Supplementary Figure 3(a) shows that there was no oxygen in the as-coated surface; however, approximately 15 at.% oxygen was observed on the wear track after the tribotest. The XPS spectra of Si 2p revealed the formation of Si–O–Si and Si–O bonds on the wear track after the ball-on-disk tribotest in 24,000 cycles (Supplementary Figure 3(b)). Thus, the low-friction tribofilm could form on the wear track, and the surface of the ball corresponding to the tribological mechanisms during the ball-on-disk tests is shown in Fig. 6c, which well matched the trends of CoF. Figure 7a, c show that the fluctuating CoF in Fig. 6a could be attributed to the formation of unstable and abrasive iron oxide debris and/or tribofilm. Furthermore, when using Si fraction greater than ~ 8 at.%, CoF was considerably lower and showed a very short running-in period because Si-rich debris and tribofilm were stably formed at the interface between the coating and ball. Finally, the abundance of debris and stable tribofilm clearly reduced CoF and its long-term stability. The further addition of Si fraction destructed the lubricating debris remaining on the surface of the ball; thereafter, CoF again showed increments.

**Figure 8:**
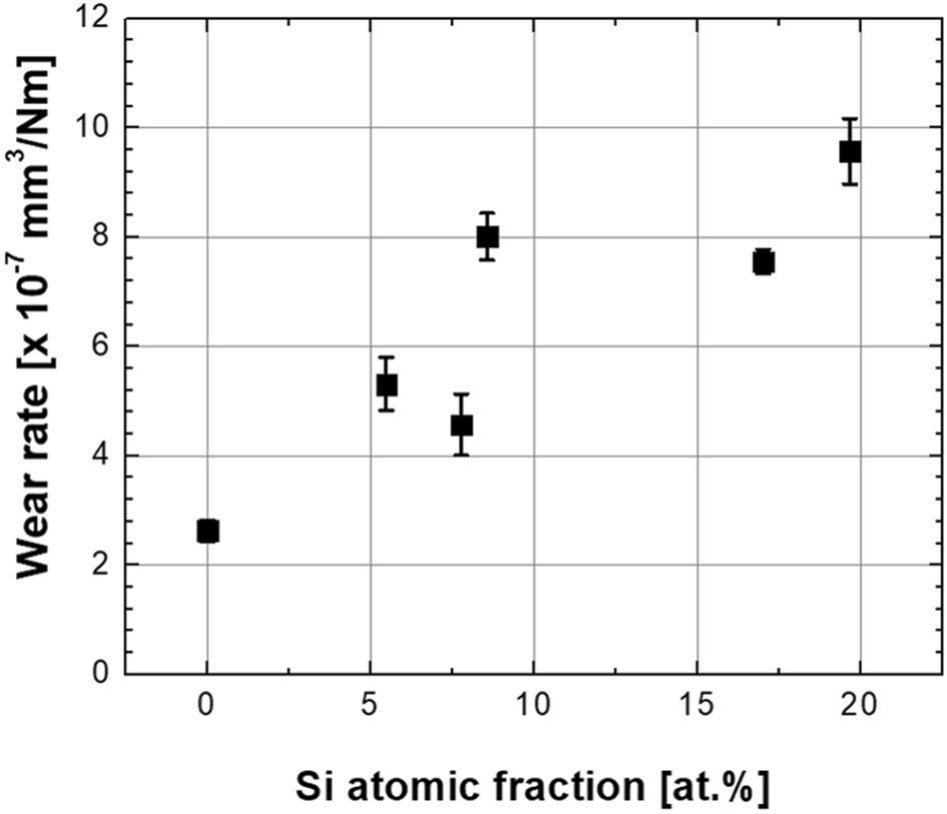
Wear rates of Si-doped DLC coatings after the tribotests (data derived from analysis of the wear tracks).

Figure 8 presents the wear rate calculated from the wear track widths and depths measured after the ball-on-disk tests. As shown in the figure, the worn amount of Si-doped DLC was higher than that on pure ta-C under the same conditions. For a small amount of Si doping (less than ~ 7 at.%), the wear rate doubled from 2.6 × 10^−7^ to 4.5 × 10^−7^ mm^3^/Nm. For a higher Si fraction (greater than ~ 8 at.%), the wear rate was ~ 60% higher than that of less Si-doped substrates. However, the wear rate remained at approximately 10^−7^ mm^3^/Nm level, which was not a very critical value considering the lowered hardness of the sample. In most cases of Si doping on DLC coatings, such as a-C:H:Si and a-C:Si, the wear rate could be increased by changing its order^45^. Conclusively, the doping of Si facilitated the effective formation of low-friction surface characteristics, and coating durability could be achieved using the optimal Si doping level without a critical increase in the wear rate.

## Conclusions

A low-friction DLC coating was fabricated using the Si doping method. The Si fraction was controlled by varying the TMS gas flow rate during the FCVA deposition of the graphite target. The microstructures were evaluated using XPS analysis, and tribological characteristics were studied via ball-on-disk tribotests. The formation of tribofilm, which could critically affect the tribological properties between two different materials, was observed via optical microscopy. The EDS and XPS spectra revealed the formation of Si-rich debris and tribofilm on the worn surfaces. The Si fraction increased with an increase in the TMS gas flow rate during the FCVA deposition. Extremely fine surface roughness could be achieved by Si doping without the formation of macroparticles on the surfaces, likely because of a higher deposition rate and reduced number of macroparticles with an increase in the TMS gas flow rate. Free-standing dangling bonds remaining after the deposition of pure ta-C coating could be terminated by the reaction of Si–C bonds with decomposed TMS gas. Increasing the Si fraction decreased the surface hardness with an increase in the sp^2^ ratio. Si atoms doped on the DLC film facilitated the formation of Si-rich debris and tribofilm; for instance, silica sol enhanced lubricating friction and yielded CoF of less than 0.05. The Si fraction of greater than ~ 8 at.% on the outermost surface effectively reduced the running-in period (under 1000 cycles) and achieved low friction. When the Si fraction exceeded the critical point (~ 20 at.%), rapid destruction of Si-rich debris and tribofilm was observed owing to a lack of moisture, humidity, and mechanical properties of the films. The optimal Si doping level could effectively enhance the tribological characteristics compared to those of steel by suppressing the formation of iron oxides and inducing the rapid formation of Si-rich debris and tribofilm. The a-C:Si:H coating for low-friction surfaces can be applied to the counter driving part of the product for protection against wear because a decrease in the mechanical properties can lead to a higher wear rate of the coating itself.

## Supplementary Information

Supplementary Information.
