# Supplementary material for: Effects of silicon doping on low-friction and high-hardness diamond-like carbon coating via filtered cathodic vacuum arc deposition
Source: Sci Rep. 2021 Feb 11;11:3529. doi: 10.1038/s41598-021-83158-4 (PMC7878482; doi:10.1038/s41598-021-83158-4)
Supplement: Supplementary file 1 — Supplementary Information. [file 41598_2021_83158_MOESM1_ESM.docx]

**Supplementary Information**

**Effects of silicon doping on low-friction and high-hardness diamond-like carbon coating via filtered cathodic vacuum arc deposition**

Jae-Il Kim^a^, Young-Jun Jang^a^, Jisoo Kim^b,c,*^, Jongkuk Kim^a,*^

^a^Department of Extreme Environmental Coatings, Surface Technology Division, Korea Institute of Materials Science (KIMS), 797 Changwondae-ro, Seongsan-gu, Changwon-si, Gyeongnam-do, 51508, Republic of Korea

^b^Department of Precision Mechanical Engineering, Kyungpook National University, 2559 Gyeongsang-daero, Sangju, Gyeongsangbuk-do, 37224, Republic of Korea.

^c^Department of Advanced Science and Technology Convergence, Kyungpook National University, 2559 Gyeongsang-daero, Sangju, Gyeongsangbuk-do, 37224, Republic of Korea.

^†^Corresponding Authors:

Dr. Jisoo Kim

Department of Precision Mechanical Engineering & Department of Advanced Science and Technology Convergence, Kyungpook National University, 2559 Gyeongsang-daero, Sangju, Gyeongsangbuk-do, 37224, Republic of Korea.

Tel: +82-54-530-1279; Fax: +82-54-530-1278

E-mail: [js.kim@knu.ac.kr](mailto:js.kim@knu.ac.kr)

ORCID: 0000-0001-9540-2800

Dr. Jongkuk Kim

Department of Extreme Environmental Coatings, Surface Technology Division, Korea Institute of Materials Science, 797, Changwon-daero, Seongsan-gu, Changwon-si, Gyeongsangnam-do 51508, Republic of Korea.

Tel: +82-55-294-9500; Fax: +82-55-280-3333

E-mail: [kjongk@kims.re.kr](mailto:yjjang@kims.re.kr)


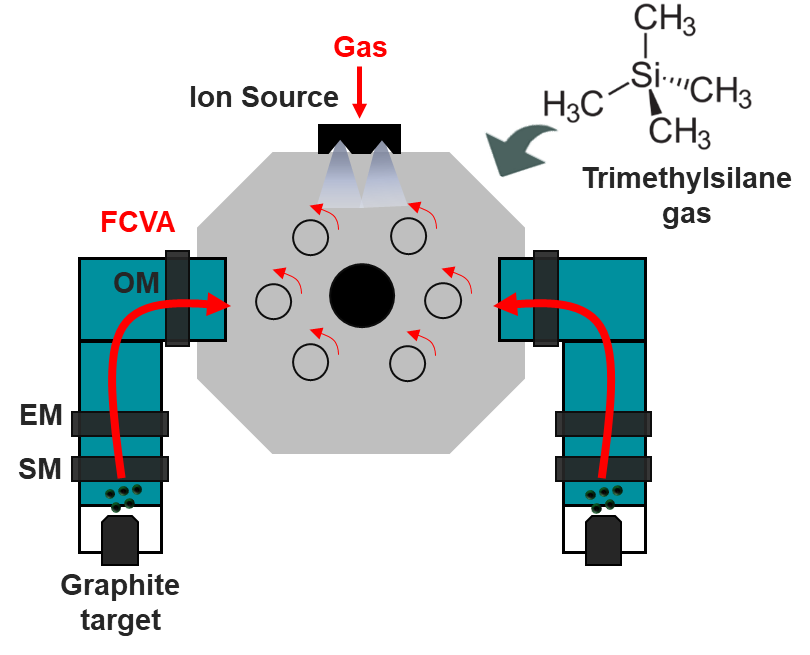


Figure S1. Schematic diagram of the experimental set up for the hybrid deposition based on filtered cathodic vacuum arc (FCVA)


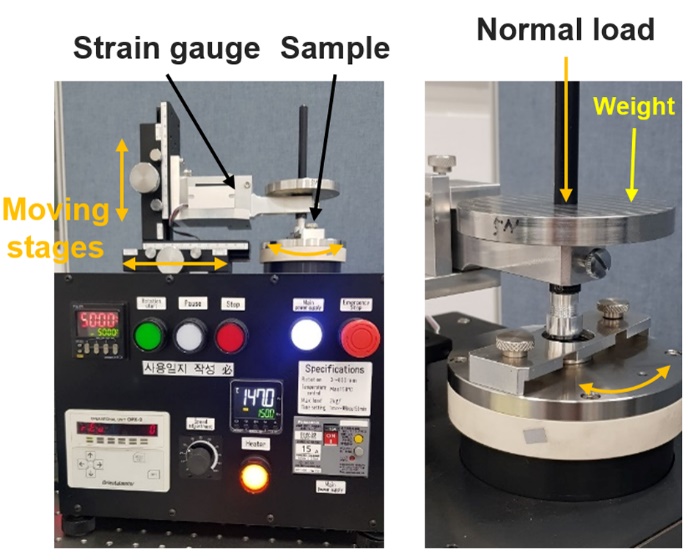


Figure S2. Experimental set up for the ball-on-disk tribotests


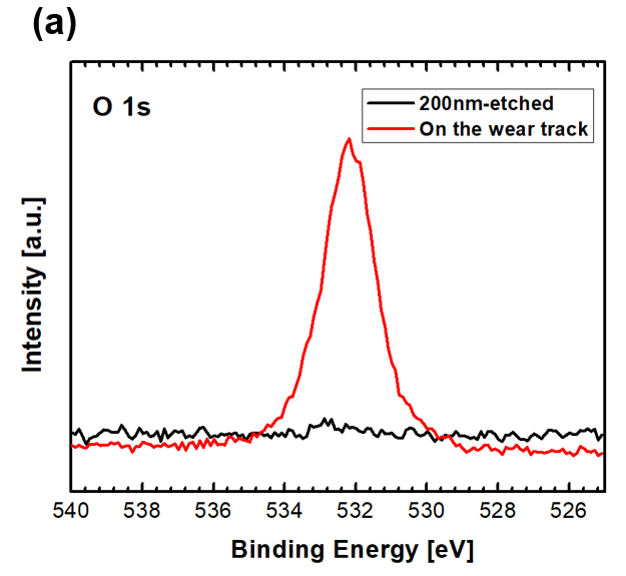


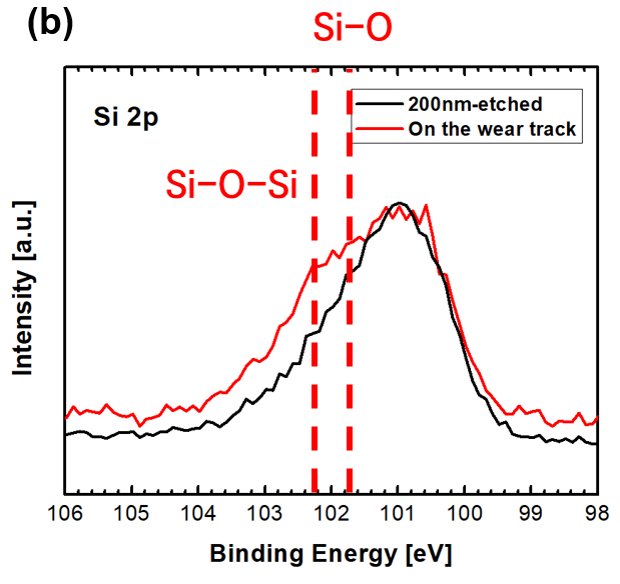


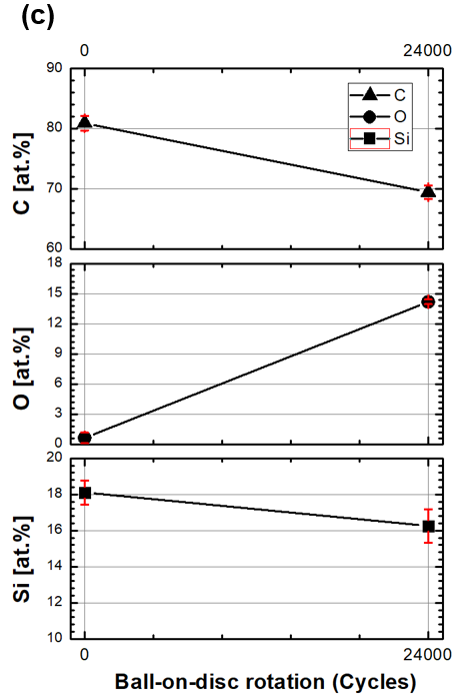


Figure S3. (a) O-1s and (b) Si-2p XPS spectra of Si-doped DLC coatings produced with the TMS flow rate of 7 sccm, and the (c) corresponding fraction of carbon, oxygen, and silicon before and after the ball-on-disk tribotest

Table S1. Detailed experimental conditions for the deposition of the pure tetrahedral amorphous carbon (ta-C) and Si-doped diamond-like carbon coating (Si-DLC)

| Coating layer | TMS flow rate  [sccm] | Deposition rate  [nm/min] | Coating time  [min] | Working pressure  [Pa] | Thickness  [μm] |
| --- | --- | --- | --- | --- | --- |
| DLC layer  (bias : -150 V) | 0.0 | 3.70 | 270 | 1.07 × 10^−2^ | ~1 |
|  | 3.0 | 6.72 | 150 | 1.87 × 10^−2^ |  |
|  | 4.6 | 8.33 | 120 | 2.93 × 10^−2^ |  |
|  | 5.4 | 9.14 | 110 | 3.73 × 10^−2^ |  |
|  | 7.0 | 10.76 | 95 | 6.80 × 10^−2^ |  |
|  | 12.0 | 15.83 | 60 | 2.00 × 10^−1^ |  |
| Etching  (bias: -75V) | Ar gas  40 | 1.05 | 60 | - | - |

Table S2. Chemical composition (%) of the SUJ2 steel ball

| Fe | C | Si | Mn | P | S | Cr | Mo |
| --- | --- | --- | --- | --- | --- | --- | --- |
| Bal. | 0.95–1.10 | 0.15–0.35 | ≤0.50 | ≤0.025 | ≤0.025 | 1.30 | ≤0.080 |
